# Fibrotic Events in the Progression of Cholestatic Liver Disease

**DOI:** 10.3390/cells10051107

**Published:** 2021-05-05

**Authors:** Hanghang Wu, Chaobo Chen, Siham Ziani, Leonard J. Nelson, Matías A. Ávila, Yulia A. Nevzorova, Francisco Javier Cubero

**Affiliations:** 1 Department of Immunology, Ophthalmology & ENT, Complutense University School of Medicine, 28040 Madrid, Spain; travelerwhh@163.com (H.W.); bobo19820106@gmail.com (C.C.); sziani@ucm.es (S.Z.); yulianev@ucm.es (Y.A.N.); 2 Health Research Institute Gregorio Marañón (IiSGM), 28007 Madrid, Spain; 3 Department of General Surgery, Wuxi Xishan People’s Hospital, Wuxi 214000, China; 4 Institute for Bioengineering (IBioE), School of Engineering, Faraday Building, The University of Edinburgh, Edinburgh EH9 3 JL, Scotland, UK; L.Nelson@ed.ac.uk; 5 Institute of Biological Chemistry, Biophysics and Bioengineering (IB3), School of Engineering and Physical Sciences (EPS), Heriot-Watt University, Edinburgh EH14 4AS, Scotland, UK; 6 Hepatology Program, Center for Applied Medical Research (CIMA), University of Navarra, 31008 Pamplona, Spain; maavila@unav.es; 7 Centro de Investigacion Biomedica en Red, Enfermedades Hepáticas y Digestivas (CIBERehd), 28029 Madrid, Spain; 8 Instituto de Investigaciones Sanitarias de Navarra IdiSNA, 31008 Pamplona, Spain; 9 Department of Internal Medicine III, University Hospital RWTH Aachen, 52074 Aachen, Germany

**Keywords:** cholangiocytes, hepatic stellate cells (HSCs), periductular fibroblasts, cholestasis, fibrosis

## Abstract

Cholestatic liver diseases including primary biliary cholangitis (PBC) and primary sclerosing cholangitis (PSC) are associated with active hepatic fibrogenesis, which can ultimately lead to the development of cirrhosis. However, the exact relationship between the development of liver fibrosis and the progression of cholestatic liver disease remains elusive. Periductular fibroblasts located around the bile ducts seem biologically different from hepatic stellate cells (HSCs). The fibrotic events in these clinical conditions appear to be related to complex crosstalk between immune/inflammatory mechanisms, cytokine signalling, and perturbed homeostasis between cholangiocytes and mesenchymal cells. Several animal models including bile duct ligation (BDL) and the Mdr2-knockout mice have improved our understanding of mechanisms underlying chronic cholestasis. In the present review, we aim to elucidate the mechanisms of fibrosis in order to help to identify potential diagnostic and therapeutic targets.

## 1. Introduction

Cholestasis is a chronic liver disease characterised by bile flow obstruction in the liver, bile acid (BA) accumulation, and increased BA concentration in the systemic circulation. Thus, during cholestasis impaired bile formation and processing with insufficient bile reaching the duodenum, leads to the accumulation of intrahepatic and systemic BAs and other potentially toxic cholephilic bacteria. The aetiology of cholestasis includes disorders of bile secretion by hepatocytes and/or biliary epithelial cells (BECs), mechanical processes (e.g., stones, tumours) destroying/blocking smaller and/or larger intrahepatic bile ducts, or immune-mediated fibrotic cholangitis, such as primary biliary cholangitis (PBC) and primary sclerosing cholangitis (PSC) [1,2]. Therefore, BA metabolism plays an important role since the obstruction of bile flow leads to cholestatic injuries, such as in PBC and PSC [3].

Therapies for these diseases are, however, limited. Although ursodeoxycholic acid (UDCA) treatment can significantly improve the prognosis of PBC patients and prolong transplant-free survival, the treatment options for those who do not respond to UDCA remain scarce. Other drugs associated with novel therapies in cholestatic diseases (PBC/PSC) are still in clinical trials, including obeticholic acid (OCA) [4], all-trans retinoic acid (ATRA) [5], Bezafibrate [6], Fenofibrate [7], etc. [8,9]. In addition, there are currently no available drugs to treat PSC [10]. Many of these disorders become chronic, therefore leading eventually to biliary cirrhosis and the need for liver transplantation [11]. Cholestatic liver disease can also cause liver failure and increase the risk of hepatocellular carcinoma (HCC) or cholangiocarcinoma (CCA) [12,13].

Several signalling pathways have been described in the development of cholestatic liver disease, such as NOTCH, IL-6 and Wnt/β-catenin pathways, transforming growth factor-β(TGF-β) and hepatocyte nuclear factors (HNFs) [14,15]. There is increasing experimental evidence that immune cells strongly contribute to cholestatic liver disease, including T cells infiltrated in the liver in surgical (bile duct ligation, BDL) and genetic (multidrug resistance gene 2, Mdr2^−/−^ knockout mice) preclinical models [16]. Patients diagnosed with PBC, displayed infiltration of different subsets of immune cells in the portal tract areas [17]. In the present review, we discuss the mechanisms of fibrosis associated with the development of cholestatic liver disease, aiming to identify potential diagnostic and therapeutic targets.

## 2. Primary Biliary Cholangitis (PBC)

PBC, formerly known as primary biliary cirrhosis, is acquired chronic cholestasis related to the autoimmune destruction of small bile ducts, causing portal vein infiltration and fibrosis. PBC is a chronic progressive disease that leads to end-stage liver disease and its related complications [18,19,20]. It can progress to biliary cirrhosis, portal hypertension, liver failure, and is associated with esophagogastric variceal bleeding, ascites, and hepatic encephalopathy [21]. PBC is a destructive lymphocytic cholangitis and specific antimitochondrial antibodies (AMAs) target specific mitochondrial autoantigens [22]. It is characterised by the progressive damage and destruction of biliary epithelial cells (BECs; also called cholangiocytes) and increased portal vein inflammation and fibrosis [23,24], with chronic histological evidence, nonsuppurative, granulomatous, and lymphocytic cholangitis [21]. Simultaneously, in association with PBC, there are also symptoms that markedly affect the quality of life, including cholestatic pruritus, Sjogren’s syndrome, abdominal discomfort, and fatigue [25,26].

Approximately 95% of PBC patients are middle-aged women [27]. Interestingly, this disease rarely affects children [28]. The reports vary worldwide from 1970 to 2014, the annual incidence ranges from 0.3 to 5.8 per 100,000, and the prevalence rates range from 1.9 to 40.2 per 100,000 individuals, respectively, due to increased incidence and improved survival [29,30,31]. From 2004 to 2014, in the United States, the prevalence of PBC increased significantly from 21.7 to 39.2 per 100,000, of which women rose from 33.5 to 57.8 per 100,000 (an increase of 72%), while the incidence rate in men increased from 7.2 to 15.4 per 100,000 (an increase of 114%) [32]. 

Risk factors for PBC include genetic factors such as the human leukocyte antigen (HLA) and non-HLA allelic variants) [33,34], as well as environmental stimuli. In addition to the regional differences in disease prevalence and family risk, the relationship between epidemiology and bacterial infection, xenobiotics, and smoking history also emphasises the importance of environmental triggers in the pathogenesis of PBC [35,36,37,38,39,40,41]. Furthermore, PBC development has also been linked to microRNA [42] and epigenetic regulation [22]. Moreover, the gut–liver axis is also involved in PBC development. Intestinal dysbacteriosis can affect the bile acid pool and regulate bile acid-activated receptors, which disturbs bile acid metabolism [43,44]. Simultaneously, several lines of evidence suggested that dysbiosis of gut microbiota can destroy the immune homeostasis, thus promoting PBC [45,46].

Population-based historical data from the UK show that about 25% of untreated “classic PBC” patients develop chronic liver failure during this period [47]. An early prospective study found that more than 50% of patients with stage I-III PBC developed histologically confirmed cirrhosis within four years [48]. As cirrhotic individuals, PBC patients may develop complications due to the chronic nature of the disease. The presence of cirrhosis, regardless of its aetiology, is a major risk factor for hepatocellular carcinoma (HCC) or cholangiocarcinoma (CCA) [13,49].

## 3. Primary Sclerosing Cholangitis (PSC)

PSC is associated with liver damage, characterised by intrahepatic or extrahepatic bile duct injury, and fibrosis of the bile ducts inside and outside the liver, resulting in strictures of the bile ducts and obstruction of bile flow. Clinical manifestations reflect the potential sequence of bile duct injury and fibrosis leading to stricture, cholestasis, and biliary cirrhosis with progressive liver dysfunction [50]. PSC is a male-dominant disease when it is associated with inflammatory bowel disease (IBD), (65–70%), with a male-female ratio of approximately 2:1 [27,51,52,53]. Epidemiological studies show that the prevalence of PSC is about 1/10,000 cases globally, while the incidence rate in northern Europe and the United States is 0.4/100,000 to 2.0/100,000 per year [54,55]. Simultaneously, the survival rate of PSC is increasing [51,56,57,58], which may be partly attributed to early diagnosis due to the application of magnetic resonance cholangiography (MRC). The clinical characteristics of newly diagnosed patients remain stable over time, while no new diagnostic methods were introduced during this period such as before fibrosis occurs [51]. 

PSC is a typical complex disease with genetic and environmental risk factors. This important genome-wide association has shown that PSC risk is associated with certain phenotypes of human leukocyte antigens (HLA), particularly HLA-DR6, HLA-DR3, and HLA-B8, suggesting the presence of autoimmune disorders in patients with PSC [59]. The risk of PSC is also associated with, at least, 23 regions of the genome [60]. At present, no clear causal environmental factor has been identified, however, the geographical distribution of the disease in northern Europe provides some clues to consider the source of environmental risk factors [61]. Indeed, differences in lifestyle, diet, and living conditions are highly regional [50]. Clinically, inflammatory bowel disease (IBD) is the strongest condition associated with PSC—approximately 70% of patients with PSC having also IBD [62,63,64].

Bacterial cholangitis, osteoporosis, liver cirrhosis, and IBD can be caused by the progression of PSC [65,66,67,68,69,70,71]. It can lead to colorectal neoplasia, pancreatic cancer, CCA, and gallbladder carcinoma [62,69,70,72,73,74]. Large population-based studies indicate that the risk of death in patients with PSC is increased fourfold, in comparison with the general population [51]. The most common causes of death associated with PSC are CCA (32%), liver failure (15%), transplant-related complications (9%), and colorectal cancer (8%), demonstrating that the increased risk of malignancy for PSC has a significant impact on life expectancy [75].

## 4. Key Hepatic Cells Involved in Disease Progression

### 4.1. Cholangiocytes and/or Biliary Epithelial Cells (BECs)

Cholangiocytes are arranged in extrahepatic and intrahepatic bile ducts and modify the bile composition from hepatic cell tubules through the intrahepatic biliary tree. During gestation, from 12 to 16 weeks, the ductal plaque replicates in the discrete region, the expression of hepatocyte markers is inhibited, and markers of commitment towards the biliary phenotype including CK7 and CK19 begin to express [76]. Modification of bile occurs through coordinated transport of water, ions, and solutes across the apical and basolateral plasma membranes of cholangiocytes. This process is mediated by hormones, nucleotides, peptides, neurotransmitters, bile acids, and other molecules, through different signalling pathways [77]. 

Cholangiocytes are a group of target cells whose malfunction causes the so-called cholangiopathies, in which cholangiocytes react to exogenous and endogenous damage [78]. Cholangiocytes can be activated by a variety of injuries, including infection, cholestasis, ischemia, and xenobiotics [79]. Activated cholangiocytes are also involved in the recruitment and crosstalk of immune cells, vascular and mesenchymal cells, as well as the development of bile duct fibrosis and CCA under chronic stimuli. Features of activated BECs/cholangiocytes include increased proliferation, fibrogenesis, and pro-inflammatory secretions [14]. Depending on the of type insult, a series of morphogenetic signals and transcription factors are also involved and/or activated, including Notch, IL-6 and Wnt/β-catenin pathways, TGF-β, and HNFs [15]. Recent studies have also shown that the SRY-box transcription factor 4 (SOX4) and SOX9 are involved in the development of primary cilia, and the normal formation, elongation, and branching of the bile duct trees [80]. These two factors cooperatively control the expression of mediators of TGF-β, Notch, and Hippo/Yes-associated protein 1 (YAP1) signalling pathways, which are required for normal biliary development. They are also related to the Wnt/β-catenin signalling pathway and are involved in liver cilia disease [81], such as polycystic liver disease (PLD) [82]. 

### 4.2. Periductular Fibroblasts and Hepatic Stellate Cells (HSCs)

Fibrosis is an abnormal wound healing reaction arising from inflammation misregulation. In this process, chronic damage to any tissue will activate myofibroblasts and produce fibrous scars [3]. Myofibroblasts are rare in normal tissues, and their source depends on the tissue and type of injury. A plethora of evidence has shown that HSCs and portal fibroblasts (PFs) are the main sources of fibrotic liver myofibroblasts [83]. However, the composition of myofibroblasts varies depending on the pathogenesis of liver fibrosis. Hepatotoxic liver fibrosis (hepatic damage) is mainly caused by activation of HSCs [84], but activated PFs (aPFs) are related to the pathogenesis of cholestatic fibrosis (obstruction of bile flow, such as in PBC and PSC [85]. PFs are ‘periductular mesenchymal cells (or fibroblasts)’ that maintain the integrity of the portal vein system [86]. As a response to biliary obstruction, portal (and/or myofibroblasts) proliferate, upregulate collagen I, α-smooth muscle actin (αSMA), TGF-β, IL-6/13, and generate activated myofibroblasts [87,88].

These activated myofibroblasts come from three main sources: hepatic stellate cells (HSCs), portal fibroblasts (PFs), and fibroblasts. Activated HSCs (aHSCs) are involved in the pathogenesis of experimental toxic liver fibrosis, such as CCl_4_-induced chronic liver disease and alcoholic liver disease [84]. PFs are mainly activated in the cholestatic liver fibrosis response in BDL or MDR2 knockout mice [85,89].

**Figure 1:**
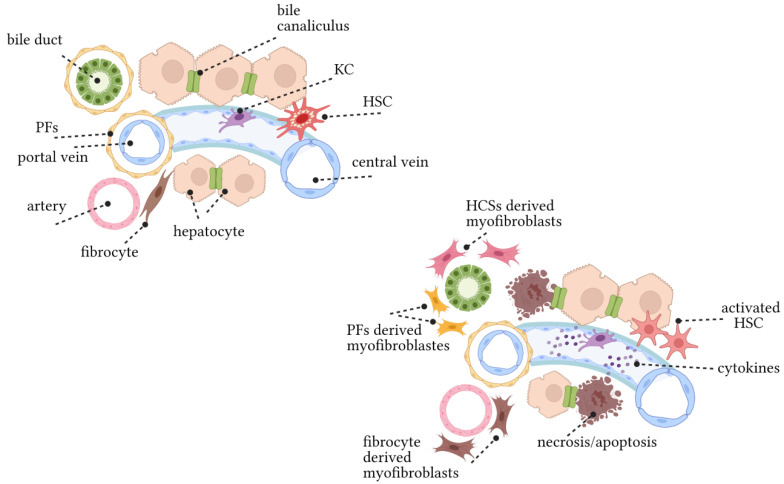
Pathogenesis of liver fibrosis. (Top left) The structure of liver lobules under physiological conditions. Biliary epithelial cells and hepatocytes, endothelial cells, HSCs, PFs, and KCs are the components of liver lobules. The bile duct, portal vein, and hepatic artery constitute the portal vein triad. HSCs are located in the space of Disse between hepatocytes and sinusoidal endothelium. HSCs are considered to be hepatic pericytes which contain lipid droplets and are the main storage cells for vitamin A. KCs are the resident liver macrophages. Only a few fibrocytes exist in a healthy liver. (Bottom right) Changes in liver lobules caused by chronic liver injury. In response to chronic liver injury, hepatocytes undergo apoptosis and release factors that recruit KCs, BM macrophages, and fibroblasts to sites of hepatic damage. KCs, macrophages, and fibroblasts release TGFβ1, which is one of the most powerful profibrogenic cytokines that can activate HSCs into collagen I-expressing myofibroblasts (e.g., αSMA). HSCs and PFs deposit ECM and a small number of fibroblasts.

The embryological origin of PFs is unknown. PFs are located around the portal vein and maintain the integrity of the bile duct and portal vein system [90]. Under physiological conditions, they are composed of a small number of fibroblasts in the liver. In response to cholestatic injury, PFs proliferate, become activated, and mediate extracellular collagen I deposition [88,91]. It is also well known that during the development of the human liver, at the ductal plaque stage, the portal mesenchyme includes αSMA-positive cells. These αSMA-positive cells are not found during the ductal plate remodelling stage, and vimentin-expressing cells begin to appear, which are believed to be PFs. It seems that PFs are derived from αSMA-positive early mesenchymal cells [83,92] (Figure 1). 

HSCs, or liver myofibroblasts, also named Ito cells, store vitamin A, and produce extracellular matrix (ECM) and collagen. HSCs play a role in the complex mechanisms of liver fibrosis and cirrhosis. They are also distributed between endothelial cells but are difficult to observe through an optical microscope [93,94]. Physiologically, quiescence HSCs (qHSCs) reside in the space of Disse (between hepatocytes and sinusoidal endothelial cells), store vitamin A and express specific markers such as glial fibrillary acidic protein (GFAP), synaptophysin, nerve growth factor p75 (NGFR, P75NTR) and lecithin retinol acyltransferase (Lrat) [3]. In response to injury, qHSCs differentiate into activated aHSCs, characterised by the expression of αSMA, become fibrogenic and mitogenic sensitive, and have the ability to actively secrete ECM proteins (especially laminin, collagen I, and III). This results in changes in the normal composition of the matrix, including cell–matrix interactions and the deposition of growth factors [95,96]. However, in response to toxic liver injury, HSCs downregulate the expression of vitamin A in lipid droplets, migrate to pericentral areas, then transdifferentiate into collagen I and αSMA positive cells, indicative of myofibroblasts [89]. Activation of HSCs has been associated with cholesterol metabolism, as previously reported [97]. Extensive experimental results demonstrated that free cholesterol could be accumulated in HSCs, as an intracellular mediator promoting HSC activation, which may contribute to a vicious cycle of HSCs activation in liver fibrosis independently of serum cholesterol levels [98,99,100] (Figure 1).

Importantly, mesenchymal cells expressing the p75NTR in the mouse foetal liver have been shown to include precursors of PFs and HSCs. The p75NTR-positive cells are located in the periphery of the liver bud, later dividing into parenchymal and portal populations, which probably reveal HSCs and PFs [101]. The portal population of p75NTR-positive cells can regulate the commitment of hepatoblasts to a biliary lineage as they express the Notch ligand JAG1 [102]. The mesodermal origin of hematopoietic stem cells and perivascular mesenchymal cells (desmin^+^, p75NTR^+^, αSMA^+^) has been demonstrated [103]. These perivascular mesenchymal cells may be the precursors of PFs, which suggests that HSCs and PFs come from a common precursor in the early embryo [83].

Specific markers for PFs include fibulin-2, IL-6, elastin, and ecto-ATPase nucleoside triphosphate diphosphohydrolase-2 (NTPD2). Useful markers to differentiate PFs from HSCs include the absence of lipid droplets and the expression of P100, α2-macroglobulin, and neuronal proteins such as the neuronal cell adhesion marker and synaptophysin [83]. The difference between aPFs and aHSCs is the expression of Thy1, Fibulin2, elastin, Gremlin1, extracellular ATPase, nucleoside triphosphate hydrolase 2, mesothelin (Msln), and mucin 16 (Muc16), as well as the lack of HSCs markers [89].

## 5. Signalling Pathways Involved in Pathogenesis

After cholangiocyte injury, infiltration of immune cells occurs, activating Notch, hedgehog (Hh), Wnt, and other signalling pathways—inducing the increase in fibroblasts due to HSCs activation, which further promote BECs proliferation. Subsequent pathological sequelae including biliary stricture formation, bile retention, bile-related toxic stress, inflammatory cells, or formation of immune cells around the bile duct further aggravate the disease.

### 5.1. Notch Signalling Pathway

In the past few years, the mechanisms and influence of Notch signalling in liver fibrosis have been developed. Rat HSCs express the Notch receptor in vitro and begin to express JAG1 after activation and differentiation into myofibroblast-like cells [104]. The expression of Notch 2/3, Hey 1/2 increases significantly in the process leading from quiescent HSCs into activated myofibroblasts [105]. High activation of the Notch signal was also observed in hepatic progenitor cells isolated from tissues with PBC [106]. In addition, the number of Notch 1/3/4-positive cells increased significantly in the fibrotic area of Chemokine (C-C motif) ligands 4 (CCl_4_)-injured rats [107]. In the CCl_4_-induced rat liver fibrosis model, Notch signalling was also hyperactivated [108]. Moreover, inhibition of Notch in CCl_4_-induced liver injury significantly damaged HSC activation and triggered the development of fibrosis, and inhibition of this pathway in the liver can prevent or ameliorate fibrosis [109].

**Figure 2:**
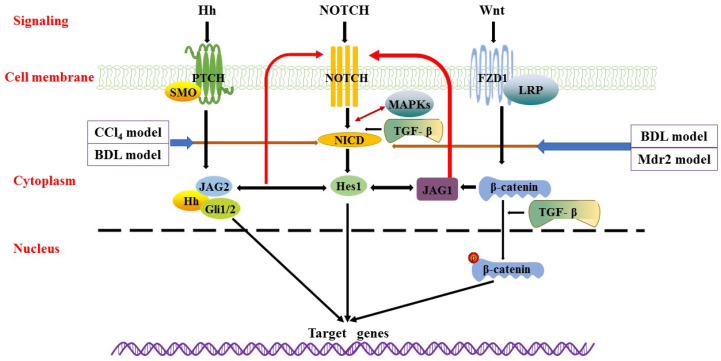
Wnt/β-catenin, Notch, and Hedgehog signalling pathways. Wnt/β-catenin signalling is transmitted through the Frizzled (FZD) receptor, thereby stabilizing β-catenin. Phosphorylated β-catenin translocated into the nucleus to regulate the expression of target genes. There is crosstalk between Wnt/β-catenin signalling and Notch/TGF-β signalling. In Notch signalling, binding of Notch ligands to the receptor results in two proteolytic cleavages to release NICD. The released NICD then translocated into the nucleus, activating transcription factors Hes1, JAG1, and JAG2, whilst the Notch signal pathway interacts with Hh and Wnt signalling pathways. In Hedgehog signalling, Hh ligand secreted by Hedgehog secretory cells binds to PTCH or SMO and generates activated Gli that translocated to the nucleus, inducing target gene expression. These main survival pathways and sophisticated interactions between signalling pathways (i.e., TGF-β and MAPKs) constitute a complex regulatory network for the survival and proliferation of BECs.

During liver fibrosis development, Notch interacts with other signalling pathways, such as TGF-β, Hippo, and Hh, whilst crosstalk between TGF-β activation and Notch occurs in liver fibrosis [110,111] (Figure 2). TGF-β in the liver may partly promote fibrosis by stimulating Notch activity in HSCs. KCs together with bone marrow-derived macrophages are thought to be the main source of TGF-β1 thus promoting the development of liver fibrosis [112,113]. Recent studies have shown that TGF-β2-induced expression of fibrosis genes in cholangiocytes and HSCs is related to the specific regulation of the Notch3 signalling pathway [114]. SOX9, a transdifferentiated biomarker of BECs specifically expressed in cholangiocytes, is also a downstream target of Notch signalling. After BDL in rats, Notch receptor activation, combined with overexpression of SOX9, enhanced BEC proliferation and induced hyper-hepatic fibrosis [102]. Recently, another study from Athwal et al. [115] demonstrated the relationship between increased SOX9 and activation of the Hippo pathway in the development of liver fibrosis. Inhibition of YAP1 in CCl_4_ and BDL-induced liver fibrosis by injection of specific YAP1-related inhibitor Verteporfin resulted in decreased expression of SOX9 in HSCs. Notch and YAP1 may activate SOX9 in different cell types, both leading to HSCs activation and fibrosis induction [115,116,117]. Noticeably, YAP1/Hippo pathways are related to the activation and amplification of ductular reactive cells (DRC) [118,119]. Additionally, during tissue repair, which normally requires cell proliferation, the Hippo pathway is often downregulated by phosphorylation of its main effectors, yes-associated protein 1 (YAP1) and transcriptional co-activator with PDZ-binding motif (TAZ) [120]. In particular, macrophage-derived TNF-related weak inducer of apoptosis kinase (TWEAK) induced expansion of progenitor cells and proliferation of bile ducts in healthy mice, while fibroblast growth factor–inducible 14 (Fn14) deficient mice or neutralisation of TWEAK prevented the expansion of progenitor cells in cholestasis mice [121,122].

### 5.2. Hedgehog (Hh) Signalling Pathway

Hh is one of the morphogenetic signalling pathways. There are three mammalian Hh proteins—Sonic hedgehog (Shh), Indian Hedgehog (Ihh), and Desert Hedgehog (Dhh). The downstream effector of Hh signalling is Gli1/2, a transcription repressor of the polycomb group and a central regulator of normal stem cell self-renewal. Regardless of the aetiology, it has been proved that the Hh signal is upregulated in the injured liver [123], and it increases with worsening of liver injury and fibrosis [124]. A previous study demonstrated liver accumulation of Hh ligands and activation of the Hh signalling pathway in the livers of BDL rodents and PBC patients [125]. Hh and Notch stimulate each other to promote HSCs activation and subsequent fibrosis [126]. In cholangiocytes, Notch promotes Shh signal transduction by regulating transport inside and outside primary cilium (PC), while Shh promotes Notch signal transduction by directly upregulating Hairy and enhancer of split-1 (Hes1) and Jagged canonical Notch ligand 2 (JAG2) [127] (Figure 2). In vitro and in vivo studies have shown that the Notch signalling pathway drives epithelial to mesenchymal transition (EMT). The interaction between Notch and Hh signalling pathways promotes the transdifferentiation of HSCs into myofibroblasts that involves an EMT, triggering fibrogenesis [108,126]. Finally, Notch also enhances the inflammatory response and M1 polarisation of macrophages [128].

### 5.3. Wnt Signalling Pathway

The Wnt signalling pathway is likely one of the main factors contributing to the progression of cholestatic liver disease. Wnt signal transduction induces liver fibrosis by promoting HSCs proliferation and activation, accompanied by ECM synthesis, EMT increase, or interaction with other fibrosis mediators [129,130]. Moreover, excessive accumulation of ECM is considered to be a key event in the pathogenesis of ageing-related liver fibrosis [131,132]. Several studies have shown that Wnt signalling is involved in the progression of liver fibrosis, and many components are upregulated and implicated in this process [133,134,135,136] (Figure 2). Conversely, the expression of some Wnt receptors (such as Frizzled 1 (FZD1)) and co-receptor low-density lipoprotein receptor-related protein5/6 (LRP5/6) in activation of HSCs increased in the progression of liver fibrosis but with decreased expression of FZD4/8 [136,137,138,139]. Furthermore, β-catenin is the main downstream effector of classical Wnt signalling, and the loss function of β-catenin will affect the metabolism of BAs. In the BDL model, complete obstruction of bile flow can lead to hepatic cholestasis. Therefore, the enhanced inhibition of BAs synthesis by inhibiting or β-catenin loss can improve the progression of cholestasis. Interestingly, Pradhan [140] showed that the knockdown of β-catenin in Mdr2^−/−^ mice resulted in increased inflammation, cell senescence, promoted fibrosis, and impaired liver regeneration after injury. In Mdr2^−/−^ mice, this phenotype is mainly driven by the toxicity of BAs lacking phospholipids. Therefore, reducing injury, improving regenerative response, and/or maintaining bile flow to prevent stasis are essential for maintaining the liver function, at least in mice.

### 5.4. Other Related Signalling Pathways

In addition to the above signalling pathways, other studies have found that the c-Jun N-terminal kinase (JNK), a member of the MAPKs family, is involved in the regulation of proliferation, cell death, inflammation, and metabolism [141,142]. JNK contributes to the activation of HSCs, induces overexpression of αSMA during the procession of liver fibrosis [143,144], and promotes the production of myofibroblasts. Kluwe et al. [144] showed that the phosphorylation of JNK increased significantly in mouse liver after BDL or CCl_4_ administration, as well as in the human fibrotic liver, mainly in fibroblasts. In vivo, the inhibition using a pan-JNK inhibitor did not affect liver injury but significantly reduced fibrosis after BDL or CCl_4_. JNK1-deficient mice showed reduced fibrosis after BDL or CCl_4_, while JNK2-deficient mice showed increased fibrosis after BDL but no change after CCl_4_. In culture, pan-JNK inhibitors prevent the activation of human HSCs induced by TGF-β, PDGF, and angiotensin II-induced murine HSCs activation, and reduce PDGF and TGF-β signal transduction. Zhao [143] showed the specific role of Jnk1 in HSC activation and ECM formation. The absence of Jnk1 correlated with a lower proliferation and survival of HSCs, demonstrating the pivotal contribution of Jnk1 in the development of liver fibrosis in HSCs. However, signalling pathways involved in JNK activation related to liver fibrosis are also participate in bidirectional crosstalk, including TNF-α and NF-κB [145]. Collectively, sustained activation of pre-fibrosis-related signalling pathways may promote the progression of liver fibrosis, combined with immune cell infiltration around biliary tracts.

## 6. Pathophysiology

### 6.1. Hepatobiliary Acid-Base Balance Mitochondria miRNAs

Although the complete pathogenesis of PBC remains elusive, the molecular mechanisms governing several stages during disease progression have been identified. The Cl^−^/HCO_3_^−^ anion exchanger 2 (AE2) mediates Cl^−^/HCO_3_^−^ exchange on the plasma membrane, regulates intracellular pH, and bile HCO_3_^−^ secretion, thus forming a bicarbonate rich ‘umbrella’ on the top surface of BECs, and protecting BECs from toxic hydrophobic BAs [146]. In cholestatic liver disease (such as PBC, PSC, cystic fibrosis-related liver disease (CFLD), and IgG4-related cholangitis (IRC), the dysregulation of the acid-base balance may be common [147]. It is noteworthy that the expression and activity of AE2 in liver and monocytes of PBC patients are decreased [148,149,150]. Therefore, it has been suggested that the reduction of the expression of AE2 leads to changes in BAs composition and may promote bile ductular injury [148,149,150]. In the AE2 knockout mouse model, IFNγ and IL-12 production were enhanced, CD8+ T cells were expanded, T regulatory cells were downregulated, whilst antimitochondrial antibodies (AMAs) and mild histological evidence of severe portal inflammation were also found [151]. Recent evidence has revealed that the imbalance of miRNAs is associated with bile duct diseases [152], especially miR506. Erice et al. [153] found that the overexpression of miR506 was associated with abnormal expression of pyruvate dehydrogenase complex (PDC-E2). MiR506 is upregulated in the intrahepatic bile duct of PBC patients, regulating the secretion of BECs by negatively targeting AE2. In addition, miR506 also regulates type III inositol triphosphate receptor (InsP3R3), an intracellular Ca^2+^ channel in the endoplasmic reticulum that positively regulates bile duct secretion [153]. Proteomic analysis of human bile duct cells showed that upregulation of miR506 affected the metabolism of proteins involved in basic biological processes, especially in mitochondria [154]. In addition, in a mitochondrial stress test, overexpression of miR506 increased mitochondrial respiration rate, and ATP production was not proportional to oxygen consumption due to proton leakage in the mitochondrial inner membrane, resulting in decreased ATP production in cholangiocytes overexpressed with miR506 [155,156]. Upregulation of miRNA506 in cholangiocytes triggered a metabolic switch, which encompassed increased glycolysis, endoplasmic reticulum-associated oxidative stress, and DNA damage [157]. Overall, upregulation of miR506 in cholangiocytes induces a more stable PBC-like phenotype. In addition to miR506, there are other miRNAs implicated in cholestatic liver diseases such as miR33a, miR122a, and miR422a.

### 6.2. Cholesterol Metabolism and Canalicular Transporters

Cholesterol 7α-hydroxylase (CYP7A1) and the canalicular bile salt export pump (BSEP) are two of several enzymes and transporters that play an important role in maintaining the bile acid reserve [158]. The microRNAs miR122a and miR422a have been reported to inhibit the translation of mRNA encoding CYP7A protein [159]. Overexpression of liver miR33, expressed from an intron of the sterol regulatory binding protein 2 (SREBP2) [160], reduces the bile acid reserve, increases liver cholesterol, and reduces cholesterol, in mouse serum. Furthermore, overexpression of CYP7A1 in mice has been shown to induce significant activation of SREBP2 regulated cholesterol metabolism. This suggests that the axis formed by CYP7A1–SREBP2–miR-33a plays an important role both in the regulation of hepatic cholesterol and in bile acid homeostasis [161,162]. Therefore, miRNAs represent an interesting target in the study of cholestatic liver diseases.

### 6.3. Immune Targets

AMAs mainly target the immunodominant PDC-E2 autoantigen which is located on the 2-oxo dehydrogenase complex (2-OADCs) in the inner membrane of mitochondria, while it only targets the small bile duct in PBC [163,164]. PDC-E2 auto reactive CD4^+^ and CD8^+^ T cells are enriched in the liver and hilar lymph nodes of patients with early and advanced diseases [165,166]. Although AMAs may not be independently pathogenic in vivo, in the presence of macrophages and BECs apolipoproteins, pro-inflammatory cytokine bursts may develop, leading to chronic biliary tract inflammation [167]. In addition to hepatic immunoregulation, innate immunity, including natural killer (NK) cells and mucosal-associated invariant T cells (MAITs), and acquired immunity affect the development of PBC. Moreover, the HLA allele is one of the genetic factors critical to changing the way of antigen presentation to determine the sensitivity of PBC, although the role of the HLA allele in the sensitivity of PBC is complex [168].

PSC is another type of autoimmune inflammatory liver disease. Although the influence of genetic and environmental factors is still unknown, our understanding of PSC genetics has made great progress in the last 10 years. As previously mentioned, a remarkable feature of PSC is that it is associated with IBD and ulcerative colitis (UC). GWAS showed that HLA loci in linkage disequilibrium [169,170] exists between IBA and PSC. Typical fibrotic cholangitis with irregular narrowing of the bile duct tree and scar formation may be mediated by HLA-restricted T cell immunity in early stages, leading to the release of profibrogenic cytokines (such as TGF-β) and triggering an inflammatory cascade. The reaction involves other cell types in the matrix around the biliary tracts, such as fibroblasts. The interaction between damaged epithelial cells and subsequently activated stromal fibroblasts leads to a characteristic fibrotic response, resulting in biliary stricture and cholestasis, which further promotes parenchymal injury. The putative PSC sensitive gene encodes the key membrane surface bile salt receptor Takeda G protein-coupled receptor 5 (TGR5) and the glycocalyx stabilizing enzyme fructosyltransferase 2 (FUT2), located in the cilia of apical cholangiocyte membranes acting as a chemosensor [171,172]. TGR5 and FUT2 can protect cholangiocytes against potentially toxic bile acids; therefore, their dysfunction may contribute to the development of chronic fibrotic cholangiopathy [173].

## 7. Mouse Models of Cholestasis Liver Disease

Previous studies with Mdr2^−/−^ mice models, a classic cholestasis model, showed that before the detectable histological and biochemical evidence of hepatic cell damage, significant hepatic neutrophil infiltration is observed after the elevation of pro-inflammatory cytokines [174]. Furthermore, compared with normal liver, there were more peribiliary M1- and M2-like monocyte-derived macrophages in the livers of Mdr2^−/−^ mice, an event also occurring in stage IV PSC patients [9]. Mdr2 has been found to be associated with cholestatic diseases. The P-glycoprotein encoded by Mdr2 plays an important role in the transport of phosphatidylcholine (PC) to the outer lobule of the tubular cell membrane, which helps to extract and excrete bile acids into bile [175]. In the Mdr2^−/−^ mouse model, mice fail to secrete PC into the bile, and bile becomes toxic due to the increased concentration of free nonmicellar BAs [176]. After the accumulation of toxic bile salts, the tight junctions and basement membranes of the bile duct are severely compromised. This leads to bile acid reflux into the portal vein, leading to nonsuppurative inflammatory cholangitis, periductal fibrosis, and ductal hyperplasia, of the ‘onion-skin’ type. In addition, atrophy and death of bile duct epithelial cells in 8-week-old mice led to the activation of fibroblasts around the duct, whilst fibrosis around the duct caused obstructive cholangitis [177].

BDL rodents are widely used experimental models to study obstructive cholestasis and proliferation of the bile ducts. In cases in which this surgical intervention induces acute obstructive jaundice with progression to cirrhosis with portal fibrosis, there is increased expression of pro-inflammatory cytokines such as TNF-α and IL-6 and of profibrotic proteins such as collage-α1 [178]. The BDL program introduces biomechanical stress into bile duct epithelium and initially triggers compensatory proliferation and expansion of BECs [179,180]. Proliferative BECs can secrete a variety of profibrotic cytokines, promote the activation and proliferation of myofibroblasts and HSCs, and promote the synthesis of excessive ECM, thus initiating the development of chronic liver fibrosis [181].

A dominant-negative form of TGF-β receptor type II (dnTGFβRII) mice is also used as a model of cholestasis. TGF-β is a cytokine that has pleiotropic roles on cell growth and differentiation. As a negative regulator of the immune system, it modulates the activation of regulatory CD4^+^ T cells [182]. In order to study the role of TGFβ1 in T cell homeostasis, dnTGFβRII mice were generated under the control of the CD4 promoter, which resulted in the specific abolition of TGFβ1 signalling in CD4-positive T cells. In these mice, the absence of TGFβ1 causes the development of autoimmune inflammatory disease in several organs related to the production of different autoreactive antibodies [183,184]. Specifically, in the liver, dnTGFβRII mice develop diverse characteristics of PBC with AMA-positive lymphoid cell infiltration of the portal spaces and elevated levels of serum cytokines such as IL-6, IFNγ, and TNFα [182]. Table 1 summarises autoantibodies in PBC and PSC.

## 8. Therapeutic Opportunities

The treatment of cholestatic diseases includes surgical treatment and drug treatment. The key point of treatment for cholestasis in the early stages is based on reducing inflammation, as monitored by laboratory (i.e., transaminases or immunoglobulin G level) or histological evaluation in order to prevent or delay fibrosis. In drug therapy, UDCA is the first-line drug in clinic. UDCA has proven beneficial in about 60% of PBC patients but is less effective in PSC [194,195,196]. Obeticholic acid (OCA) is a synthetic derivative with anti-fibrotic effects [197]. Several OCA tests on PBC patients with insufficient UDCA reaction demonstrated that OCA can effectively improve the symptoms of patients. Currently, there is no drug therapy for PSC [5,198]. With the latest progress in molecular biochemistry especially related to bile acid regulation and the understanding of immune pathways, new drug therapies have emerged. Table 2 summarises new therapies in the treatment of PBS and PSC. In advanced stages of cholestasis, the main surgical treatment is liver transplantation, which is suitable for patients with advanced cirrhosis.

## 9. Conclusions

Cholestatic liver disease is characterised by impaired bile formation and transport, and insufficient bile reaching the duodenum, which, in turn, leads to the accumulation of BAs and other potentially toxic cholephilic bacteria inside and outside of the liver. PBC and PSC are common immune diseases that cause cholestatic cholangitis. Since the molecular mechanisms of cholestatic liver disease (such as PBC and PSC) are not fully understood, treatment options for cholestasis are limited. Even though short-term medication or use of normal drugs (e.g., OCA) can improve symptoms, they fail to halt disease progression. Although stimulation of bile secretion and adaptive mechanisms may reduce liver damage, in patients diagnosed with cholestatic liver disease, ultimately, liver transplantation is the only option to prolong life. Thus far, immune mechanisms targeting the pathogenesis of PBC and PSC have so far proved disappointing. In this review, we discussed the early initiation and developmental mechanisms of cholestatic liver disease combined with our current understanding of disease pathogenesis. Early activation of periductular fibroblasts, accompanied by inflammatory cytokines/factors around the bile duct, may be an important trigger for the inflammatory response that causes bile duct obstruction, involving HSCs and PFs, which are the main sources of fibrotic liver myofibroblasts. Furthermore, activation of periductular fibroblast-related signalling pathways, in turn, trigger the proliferation of myofibroblasts, collagen I deposition, and promotion of fibrogenesis, eventually leading to liver fibrosis and/or even irreversible liver cirrhosis. Therefore, if we can elucidate the pathological and molecular transduction pattern of cholestatic liver disease development and ascertain key events of the specific blockade that occurs, it could generate significant breakthroughs and allow progress in the diagnosis and treatment of cholestatic liver disease.

## Figures and Tables

**Table 1:** Autoantibodies in PBC and PSC.

Autoantibody	Target	Found Positive in Liver Disease
AMAs	PDC-E2	PBC [185,186]
	OGDC-E2	
	BCOADC-E2	
	E3BP	
ANAs	gp210	PBC and PSC [21,187,188]
	p62	
	sp100	
	PML	
Anti-Kelch	KLHL12	PBC [189]
Anti-ASGPR	ASGPR	PBC [190]
Anti-hexokinase	HK1	PBC [191]
p-ANCA/p-ANNA	Unclear	PSC [192]
Anti-GP2 IgA	Gp2	PSC [193]

AMAs, anti-mitochondrial antibodies; ANAs, antinuclear antibodies; OGDC-E2, 2-oxoglutarate dehydrogenase complex; BCOADC, branched-chain 2-oxo-acid dehydrogenase complex; E3BP, E3-binding protein; gp210, glycoprotein 210; sp100, nuclear body speckled 100 kDa; PML, promyelocytic leukaemia; KLHL12, Kelch-like 12; ASGPR, asialoglycoprotein receptor; HK1, hexokinase 1; p-ANCA, perinuclear antineutrophil cytoplasmic antibodies; p-ANNA, atypical p-ANCA; Gp2, Glycoprotein 2.

**Table 2:** Targets and drugs investigated for the treatment of PBS and PCS.

Drug	Mechanism of Action	Reference and NCT Number	
		PBS	PCS
	Agonist (Target)		
Fenofibrate	PPARα	Levy et al. [199] NCT00575042	Ghonem et al. [200] NCT01142323
Bezafibrate	Pan PPAR	Corpechot et al. [201] NCT01654731	Elsemieke et al. [202] NCT02701166
Fenofibrate and bezafibrate	PPARα		Lemoinne et al. [203]
Seladelpar	Selective PPARδ	Hirschfield et al. [204] NCT02955602	NCT04024813
Elafibranor	PPARα	Schattenberg et al. [205] NCT03124108	
Saroglitazar	PPARα and PPARγ	Lindor et al. [206] NCT03112681	
Cilofexor	FXR	NCT02943447	Trauner et al. [207] NCT02943460
Tropifexor	FXR	NCT02516605	
EDP-305	FXR	Goldstein et al. [208] NCT03394924	
Etrasimod	S1PR1 and S1PR4	NCT03155932	
	Inhibitor (Target)		
GKT137831	NOX1 and NOX4	Goldstein et al. [208] NCT03226067	
Baricitinib	JAK1 and JAK2	NCT03742973	
	Monoclonal antibody against (Target)		
Ustekinumab	IL-12 and IL-23	Hirschfield et al. [9] NCT01389973	
Abatacept	CD80 and CD86 interferes with T-cell activation	Wagner et al. [209] NCT02078882	
Rituximab	CD20 B-cell depletion	Khanna et al. [210] NCT02376335	
Infliximab	TNF-α		Hommes et al. [8]
Simtuzumab	LOXL2		Muir et al. [211] NCT01672853
E6011	CX3CL1 (fractalkine)	Goldstein et al. [208] NCT03092765	
	Antibiotics Gut microbiome: antimicrobial and immunomodulation		
Vancomycin		Ali et al. [212] NCT01322386	Ali et al. [212] NCT01802073
Vancomycin and metronidazole			Tabibian et al. [213] NCT01085760
Rifaximin			Tabibian et al. [214] NCT01695174
Probiotics		NCT03521297	
ATRA	Permissive activator of the nuclear receptor FXR/RXR		Assis et al. [215] NCT01456468
NGM282	FGF 19 analogue	Gochanour et al. [216] NCT02026401	Hirschfield et al. [217] NCT02704364

PPAR-α, peroxisome proliferator-activated receptor alpha; FXR, *farnesoid X receptor;* S1PR1, *Sphingosine-1-phosphate receptor 1;* S1PR4, *Sphingosine-1-phosphate receptor 4;* NOX1,NADPH oxidase 1; NOX4,NADPH oxidase 4; IL-12, interleukin 12; IL-23, interleukin 23; CD80, cluster of differentiation 80; CD86,cluster of differentiation 86; CD20,cluster of differentiation 20; TNF-α, tumour necrosis factor alpha; LOXL2, lysyl oxidase-like 2; CX3CL1,C-X3-C motif chemokine ligand 1; ATRA, all-trans retinoic acid; FGF 19, fibroblast growth factor 19.

## Abbreviations

2-OADCs	2-oxo dehydrogenase complex
AE2	anion exchanger 2
AMAs	antimitochondrial antibodies
ATRA	all-trans retinoic acid
Bas	bile acids
BDL	bile duct ligation
BECs	biliary epithelial cells
CCA	Cholangiocarcinoma
CCl_4_	C-C Motif Chemokine Ligand 4
dnTGFβRII	dominant-negative form of TGF-β receptor type II
ECM	secrete extracellular matrix
EMT	epithelial-to-mesenchymal transition
FUT2	glycocalyx stabilising enzyme fucosyltransferase 2
UDCA	ursodeoxycholic acid
FZD1	Frizzled 1
GWAS	genome-wide association study
HCC	hepatocellular carcinoma
Hes1	hairy and enhancer of split-1
Hh	Hedgehog
HLA	*human leukocyte antigen*
HNFs	hepatocyte nuclear factors
HSCs	hepatic stellate cells
IBD	inflammatory bowel disease
IFNg	*interferon gamma*
IL-12	interleukin 12
IL-6	interleukin 6
JAG2	jagged canonical notch ligand 2
JNKs	c-Jun N-terminal kinases
LRP5/6	low-density lipoprotein receptor-related protein5/6
MAIT	mucosal-associated invariant T cells
MRC	magnetic resonance cholangiography
NF-κB	nuclear factor kappa-light-chain-enhancer of activated B cells
NTPD2	ecto-ATPase nucleoside triphosphate diphosphohydrolase-2
OCA	obeticholic acid
PBC	primary biliary cholangitis
PC	primary cilium
PDC-E2	pyruvate dehydrogenase complex
PFs	portal fibroblasts
PLD	polycystic liver disease
PSC	primary sclerosing cholangitis
p75NTR	p75 neurotrophin receptor
SOX4	SRY-box transcription factor 4
SOX9	SRY-box transcription factor 9
TGF-β	transforming growth factor beta
TGR5	Takeda G protein-coupled receptor 5
TNF-α	tumour necrosis factor-α
UDCA	ursodeoxycholic acid
YAP1	Yes-associated protein 1
αSMA	α-smooth muscle actin
